# The decrease in Rad51 and DNA ligase IV nuclear protein expression in Msh2 knockdown HC11 cells induced the low CRISPR/Cas9-mediated knock-in efficiency at the β-casein gene locus

**DOI:** 10.5713/ab.24.0206

**Published:** 2024-10-24

**Authors:** Ga-Yeon Kim, Man-Jong Kang

**Affiliations:** 1 Department of Animal Science, Chonnam National University, Gwangju 61186, Korea

**Keywords:** Clustered Regularly Interspaced Short Palindromic Repeats/CRISPR-Associated 9 (CRISPR/Cas9), DNA Mismatch Repair (MMR), Homologous Recombination (HR), Non-Homologous End Joining (NHEJ)

## Abstract

**Objective:**

Successful gene editing technology is crucial in molecular biology and related fields. An essential part of an efficient knock-in system is increasing homologous recombination (HR) efficiency in the double-strand break (DSB) repair pathways. Interestingly, HR is closely related to the DNA mismatch repair (MMR) pathway, whereby MMR-related gene *Msh2* recognizes a mismatch of nucleotides in recombinant intermediates or gene conversion formed during HR. This study aimed to investigate how the knockdown of *Msh2* affects HR-mediated knock-in efficiency at the mouse β-casein locus. Therefore, we investigated the effect of inhibiting *Msh2* expression on the expression of the HR-related gene *Rad51* and the key enzyme DNA ligase IV involved in non-homologous end joining (NHEJ).

**Methods:**

The knock-in vector targeting the mouse *β-casein* gene locus, programmed guide RNA, and Msh2 siRNA expression vector were co-transfected in HC11 cells, or only the Msh2 siRNA expression vector was transfected. Knock-in efficiency was confirmed by polymerase chain reaction (PCR). The mRNA and protein expression of *Msh2*, HR-related gene *Rad51*, and NHEJ-related gene *DNA ligase IV* were evaluated by quantitative reverse transcription PCR (RT-qPCR) and Western blot analysis.

**Results:**

The knock-in vector efficiency at the mouse *β-casein* gene locus significantly decreased upon *Msh2* knockdown in HC11 mouse mammary epithelial cells (HC11 cell). Additionally, the knockdown of the DNA MMR-related gene Msh2 protein significantly downregulated the nuclear protein expression of the HR-related *Rad51* and NHEJ-related *DNA ligase IV* genes.

**Conclusion:**

The decreased Msh2 protein expression in the nucleus downregulated the Rad51 and ligase IV protein expressions. Consequently, reduced Rad51 expression results in decreased knock-in efficiency.

## INTRODUCTION

Gene editing is a technique capable of deleting, inserting, and substituting DNA bases. It is induced by targeted double-stranded breaks (DSBs) and endogenous cell repair pathways [1]. Gene editing has been made possible using gene scissors, first-generation zinc-finger nucleases, second-generation transcription activator-like effector nucleases, and the next-generation, clustered regularly interspaced short palindromic repeats (CRISPR)-associated protein (Cas9) [1,2].

The Cas9 endonuclease targets and cleaves dsDNA sequences of interest using the protospacer adjacent motif sequence of programmed guide RNA [3]. The main repair pathways for DSB are known as non-homologous end joining (NHEJ) and homologous recombination (HR), whereby HR is the significant pathway involved in DSB repair because it restores lost genetic information using homologous templates of sister chromatin [4,5]. Therefore, HR-mediated repair is suitable for knock-in procedures, which insert the desired sequence into the target locus by recombining an exogenously supplied DNA ‘donor template’ [6].

However, HR activity is limited to only the S and G2 phases of the cell cycle, meaning the efficiency of the HR repair pathway is extremely low [7]. Notably, a study reported an increase in HR efficiency up to 5-fold by inhibiting the NHEJ-related *DNA ligase IV* and *Ku70* genes [8]. Likewise, a study reported that Scr7 treatment in mouse embryos increased the efficiency of HDR-mediated genome editing up to 19-fold [9]. In addition, studies have reported an increase of up to 2-fold in the knock-in efficiency of fluorescent cassette donor DNA by overexpressing the HR-related gene *Rad51* [10,11].

However, from 2014 to 2018, HR-mediated knock-in efficiency was reported to be less than 20%, meaning the success rate of gene editing was very low [12]. In particular, the knock-in efficiency of large DNA fragments in gene editing was very low and reported to be a major barrier [13,14]. Therefore, further improvement in the efficiency and accuracy of the HR pathway is considered necessary for efficient gene editing.

HR and DNA mismatch repair (MMR) are major pathways in DNA replication and are closely related. For instance, it has been reported that the loss of Msh2 in yeast improves the efficiency of HR, which is the recombination between divergent sequences and HR [15]. Similarly, a study reported that Rad51 expression was enhanced in Msh2-deficient murine ES cells [16]. Moreover, Msh2 is required in the early stages of DNA repair and is reported to prevent foreign DNA from assimilating into chromosomes [17]. MMR has been reported to inhibit recombination, especially when excessive inclusion of mismatched nucleotides occurs during HR [18]. In addition, it is reported that treating bovine cells with the MMR inhibitor CdCl2 did not improve the knock-in efficiency [19]. Furthermore, Kim et al [20] reported that high-dose treatment of mouse cells with CdCl2 reduced the expression of MMR-related genes and *Rad51*, resulting in a 10% decrease in knock-in efficiency. The mechanism through which MMR increases or decreases HR efficiency in the HR repair pathway needs to be clarified.

Therefore, this study aimed to clarify whether inhibition of the *Msh2* gene increases or decreases HR-mediated knock-in efficiency using the CRISPR/Cas9 system at the *β-casein* gene locus in HC11 cells.

## MATERIALS AND METHODS

### Construction of the *Msh2* siRNA expression and knock-in vectors

In this study, we used the knock-in vector previously reported by Kim et al [20] to express erythropoietin at the mouse β-casein gene exon 3 locus. To construct the Msh2 siRNA expression vector, the siRNA sequence targeting the *Msh2* gene (GenBank accession no. NM_008628.3) was designed using the Invitrogen Block-iT RNAi designer program. The primer sequences for the Msh2 siRNA expression vector are provided in Table 1. The conditions for oligonucleotide annealing are as follows. The annealing condition was initiated at 95°C for 5 minutes, followed by a gradual temperature decrease from 95°C to 85°C at a rate of −2°C/second. Subsequently, a further reduction in temperature from 85°C to 25°C was performed at a rate of 0.1°C/second. The annealed oligonucleotides were cloned into the pSilencer 4.1-CMV puro-expression vector (Ambion Inc., Austin, TX, USA) using BamHI and HindIII restriction sites. The secured sequence in the Msh2 siRNA expression vector was confirmed according to the manufacturer’s manual. The pSilencer 4.1-CMV puro-expression control vector contained scrambled siRNA and was used as the negative control (Life Technologies, Gaithersburg, MD, USA).

### Cell culture and transfection

HC11 cells were cultured according to the culture method reported by Kim et al [20]. HC11 cells were seeded at a density of 1.4×10^5^ cells in 12 plates (SPL, Pocheon, Korea) and 2.75×10^5^ cells in 6-well plates (SPL) and incubated at 37°C and 5% CO_2_. Before transfection, the culture medium was changed to 0.5 or 1 mL of the fresh medium. Then, a 3 μg Msh2 siRNA expression vector was transfected according to the manufacturer’s manual using an Xfect transfection reagent (Takara, Tokyo, Japan). In addition, the knock-in vector (3.75 μg), pGuide-it-Zs Green1_sgRNA expression vector (1.875 μg), and Msh2 siRNA expression vector (1.875 μg) were co-transfected into the HC11 cells.

### Analysis of knock-in efficiency by polymerase chain reaction

To analyze the knock-in efficiency of the mouse β-casein gene exon 3 locus, genomic DNA was isolated from the cells and analyzed using first and second polymerase chain reaction (PCR). The knock-in efficiency was verified using the method previously described by Kim et al [20]. The first PCR was performed using the mβC5 CV S5 primer for regions outside the 5’-arm and the mβC3 GST9 (Table 1) for the GST region using the amplification reagent Solg 2X Multiplex (Solgent, Daejeon, Korea). The PCR consisted of 25 cycles of amplification at 95°C for 20 seconds, annealing at 62°C for 40 seconds, and strand extension at 72°C for 2 minutes. Subsequently, a nested PCR was performed using the mβC5 CV primer and the mβC3 GST AS7 primer using the amplification regent KOD FX Neo (Toyobo, Tokyo, Japan) and the following steps: 25 cycles of denaturation at 95°C for 20 seconds, annealing at 64°C for 30 seconds, and strand extension at 72°C for 2 minutes; followed by a final extension step of 72°C for 5 min. The PCR fragments were confirmed through electrophoresis on a 0.8% agarose gel. For comparative quantification, each DNA band was normalized to the mβCE7 region.

### Quantitative reverse transcription polymerase chain reaction

Quantitative PCR (qPCR) was performed to measure the mRNA expression of the DNA MMR-related gene *Msh2*, HR-related gene *Rad51*, and NHEJ-related gene *DNA ligase IV* in RNA isolated from HC11 cells using the method previously described by Kim et al [20]. qPCR was performed under the following conditions: Total volume of 20 μL, containing 20 ng of cDNA, 10 pmol of forward and reverse primer pairs, and 10 μL of TOPreal qPCR 2X PreMIX (Enzynomics, Daejeon, Korea). All qPCR experiments were performed in triplicate using the Mx3000p instrument (Agilent Technologies, Santa Clara, CA, USA) through the following steps: denaturation at 95°C for 10 minutes, followed by 40 cycles of amplification at 95°C for 10 seconds, annealing at 60°C for 15 seconds, and strand extension at 72°C for 15 seconds.

### Western blot analysis

Nuclear and cytoplasmic proteins isolated from HC11 cells were extracted according to the method reported by Kim et al [20]. Subsequently, 12 μg of nuclear and cytoplasmic proteins were separated using 10% sodium dodecyl sulfate-polyacrylamide gel electrophoresis and transferred to polyvinylidene fluoride membranes (Bio-Rad Co., Hercules, CA, USA). Msh2, Rad51, ligase IV, and β-actin analyses were conducted following the protocol reported by Kim et al [20]. Additionally, incubation at 4°C overnight was performed with a mouse polyclonal anti-Hdac2 antibody (dilution 1:1,000, sc-55541; Santa Cruz Biotechnology, Dallas, TX, USA). After incubating overnight, the membranes were washed with tris buffered saline with tween (TBST) and incubated with horseradish peroxidase (HRP)-conjugated secondary antibodies: Mouse immunoglobulin G (IgG) kappa binding protein (m-IgG_k_ BP) conjugated to HRP (dilution 1:2,000, sc516102; Santa Cruz Biotechnology) at room temperature for 2 hours. The membranes were washed with TBST. Msh2, Rad51, DNA ligase IV, Hdac2, and β-actin protein bands were detected using the EZ-Western Lumi Pico Kit and EZ-Western Lumi Femto Kit (Dogen, Seoul, Korea). Rad51, Msh2, and DNA ligase IV nuclear and cytosol protein bands were normalized to Hdac2 and β-actin for comparative quantification of protein levels.

### Statistical analysis

Densitometric quantification of DNA or protein bands was performed by analyzing the data using UN-SCAN-IT gel Analysis Software (Silk Scientific Inc., Orem, UT, USA). Subsequently, a statistical analysis of the mRNA levels and densitometric quantification of DNA and protein bands was performed using GraphPad Prism 5 (GraphPad Software Inc., San Diego, CA, USA). The data were analyzed in two ways. First, the data were analyzed using one-way. ANOVA, followed by the Dunnett test, to compare all columns to the control. Second, a t-test was performed to compare the differences between the two columns. A confidence interval was set at 95%.

## RESULTS

### The effect of Msh2 silencing by the Msh2 siRNA expression vector

**Figure 1:**
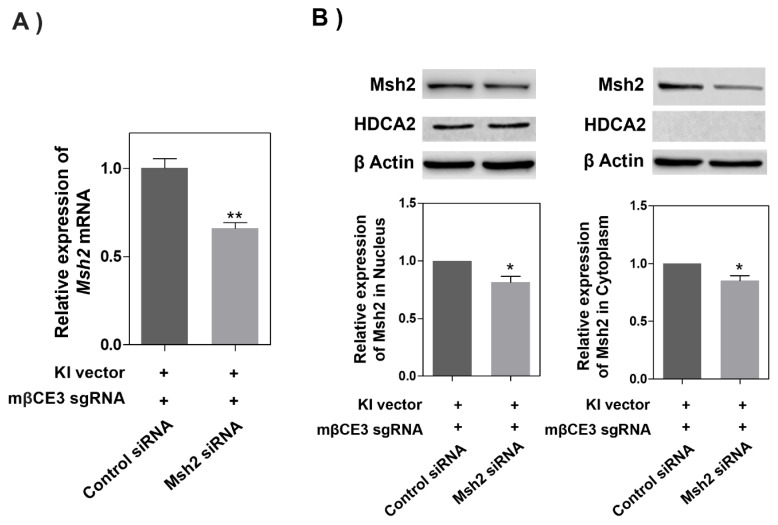
Msh2 mRNA A) and protein B) expressions in HC11 cells following transfection with the Msh2 siRNA expression vector, knock-in vector, and sgRNA expression vector. Densitometric analysis of Western blot results is presented in B) right panel. western blot results were presented in the nucleus and cytoplasm using image processing software. Western blots were normalized to Hdac2 in the nucleus and β-actin in the cytoplasm. Values are mean ± standard error of the mean from at least three independent experiments. * p<0.05, ** p<0.01.

The mRNA and protein knockdown effects of the Msh2 gene in HC11 cells transfected with the Msh2 siRNA expression vector, knock-in vector, and sgRNA expression vector were evaluated by quantitative reverse transcription PCR (RT-qPCR) and Western blot. From RT-qPCR, the *Msh2* mRNA expression was significantly reduced by about 34% in the Msh2 siRNA expression vector treatment group compared to the control group (p<0.01) (Figure 1A). The nuclear protein of Msh2 was significantly decreased by about 20% and the cytoplasmic protein was significantly decreased by about 16% (p<0.05) (Figure 1B).

### Analysis of knock-in efficiency in HC11 cells with transient *Msh2* gene knockdown

**Figure 2:**
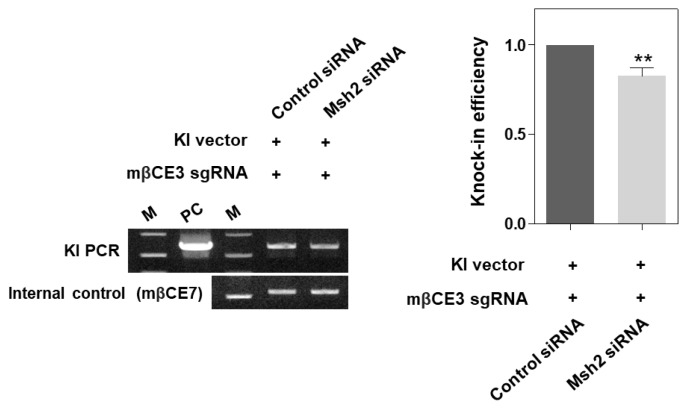
Decrease in CRISPR/Cas9-mediated knock-in efficiency in HC11 cells following Msh2 knockdown. Agarose gel electrophoresis of the knock-in DNA band is presented in the left panel. PCR results were normalized to internal control (mβCE7), and densitometric analysis of PCR results is presented in the right panel. KI, knock-in; M, DNA size marker (1 kb ladder); PC, positive control. ** p<0.01.

We evaluated knock-in efficiency at the *β-casein* gene locus in HC11 cells, where the *Msh2* gene expression was transiently reduced. The vector knock-in efficiency at the *β-casein* gene locus decreased significantly by 17% compared to the control (p<0.01) (Figure 2). As a result, it was confirmed that when the mRNA and protein expression of Msh2 decreased, the knock-in efficiency of the vector at the exon 3 locus of the *β-casein* gene decreased.

### *Msh2*, *Rad51*, and *DNA ligase IV* mRNA expressions in HC11 cells following transient *Msh2* knockdown

**Figure 3:**
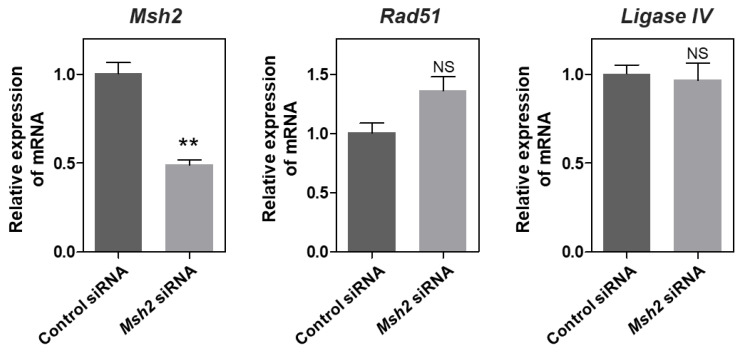
mRNA expression of *Msh2*, HR-related gene *Rad51*, and NHEJ-related *DNA ligase IV* in HC11 cells following Msh2 knockdown. NS, no statistical difference. ** p<0.01.

To investigate whether the transient knockdown of *Msh2* mRNA expression in HC11 cells affects the HR and NHEJ DSBs repair pathways, we evaluated the mRNA expression of the HR-related gene *Rad51* and the NHEJ-related gene *DNA ligase IV*. The mRNA expression of *Rad51* and *ligase IV* was evaluated in HC11 cells, in which the mRNA expression of *Msh2* was significantly reduced by approximately 50% (p<0.01) (Figure 3). Subsequently, *Msh2* mRNA knockdown did not significantly affect the mRNA expression of *Rad51* and *ligase IV* (Figure 3).

### Msh2, Rad51, and DNA ligase IV protein expressions in HC11 cells following transient Msh2 protein knockdown

**Figure 4:**
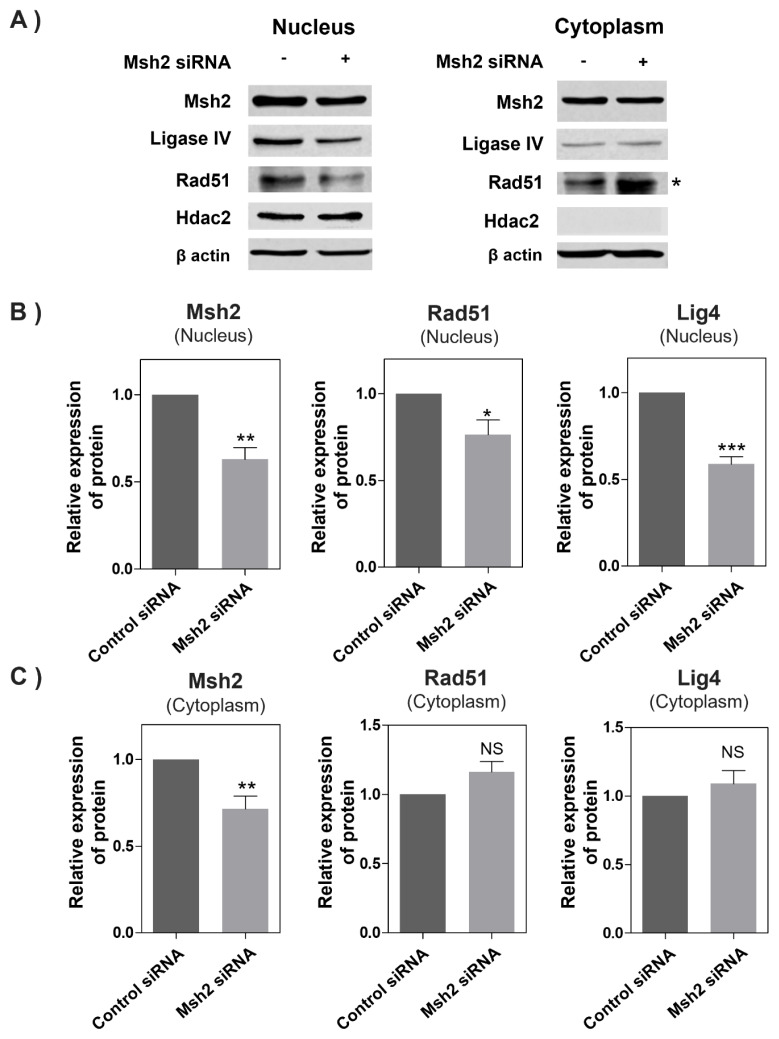
Msh2, DNA ligase IV, and Rad51 protein levels in HC11 cells following Msh2 knockdown. A) Western blot analysis of nuclear and cytoplasmic protein levels for Msh2, NHEJ-related gene DNA ligase IV, and HR-related gene Rad51 (n = 3). The densitometric analysis of Western blot results is presented in B) nucleus and C) cytoplasm. Western blots were normalized to Hdac2 in the nucleus and β-actin in the cytoplasm. NS, no statistical difference. ‘*’ indicates cytoplasmic Rad51 band. Values are mean ± standard error of the mean from at least three independent experiments. * p<0.05, ** p<0.01, *** p<0.0001.

Rad51 and DNA ligase IV protein expressions were investigated in HC11 cells where the Msh2 protein was transiently knocked down. First, the Msh2 siRNA expression vector treatment group showed that the Msh2 protein expression was reduced by about 40% (p<0.01) in the nucleus and by about 30% (p<0.01) in the cytoplasm compared to the control group (Figures 4A–4C). The Rad51 nuclear protein expression was reduced by approximately 18% (p<0.05) and DNA ligase IV by approximately 38% (p<0.0001) in the group treated with the Msh2 siRNA expression vector (Figures 4A, 4B). However, no statistically significant difference was found in the cytoplasmic protein expression of Rad51 and DNA ligase IV from the change in Msh2 expression (Figures 4A, 4C). Thus, decreasing the nuclear protein expression of Msh2 was shown to reduce the nuclear expression of HR-related proteins Rad51 and NHEJ-related DNA ligase IV, suggesting a close relationship between the expressions of Msh2, Rad51, and DNA ligase IV.

## DISCUSSION

In this study, we investigated the effect of Msh2 gene knockdown on HR-mediated gene editing at the *β-casein* gene locus in HC11 cells. Consequently, our findings show that reducing the expression of the *Msh2* gene downregulates the expression of Rad51, reducing knock-in efficiency.

The major DNA double-strand break (DSB) pathways include HR and NHEJ. In HR, Rad51 finds homology and induces strand invasion, while in NHEJ, DNA ligase IV induces ligation of the cleaved terminal strand [21]. During the HR repair pathway, Msh2, an MMR-related protein, forms a heterodimer with Msh3 or Msh6, recognizes the mismatch of recombinant intermediates, and inhibits recombination between non-identical sequences. Specifically, MutS β (Msh2-Msh3) has been reported to participate in the terminal resection of non-identical DNA 3’-ends in the strand annealing phase of HR-mediated DSBs [22]. The MMR pathway corrects nucleotide mismatches that occur during HR, thereby maintaining genome stability [23]. Therefore, excessive mismatched nucleotides can induce the anti-recombination or heteroduplex rejection pathway [24]. Furthermore, gene editing using the DNA repair pathways may form heteroduplex molecules between the target vector and genetic DNA [25]. A related study on MMR repair in the HR process reported that recombination efficiency between divergent sequences is improved in MMR-deficient cells, and gene editing efficiency is improved [26].

Thus, this study was necessary to confirm whether inhibiting Msh2 increases or decreases HR-mediated gene editing efficiency. It was confirmed that gene editing efficiency was reduced in HC11 cells when the Msh2 gene was suppressed. These results are consistent with previous reports that treatment with the MMR inhibitor CdCl_2_ reduces HR-mediated gene editing efficiency [20]. However, in previous studies, it was unclear whether Rad51 expression was decreased by CdCl_2_ or MMR inhibition, making it difficult to determine whether MMR inhibition was the cause of the reduced gene editing efficiency [19,20].

We decided to check the RNA and protein expressions of the key HR-related factor Rad51 and the critical NHEJ-related factor DNA ligase IV to clarify why Msh2 gene inhibition reduces gene editing efficiency. Since the expression of Rad51 and DNA ligase IV may be transiently increased in DSB-induced cells, the expression of Rad51 and DNA ligase IV, according to the effect of Msh2 gene inhibition, was confirmed in normal cells that do not stimulate DNA repair. Msh2 gene suppression did not change the Rad51 and DNA ligase IV RNA expressions. Further, we confirmed that as the nuclear protein expression of the Msh2 gene decreased, the nuclear protein expression of Rad51 and DNA ligase IV was also reduced. This result is similar to a study in which Rad51 foci decreased in irradiated primary human cells following the knockdown of Msh2 [27]. A similar study on MMR and HR reported that removing the MMR-related gene Msh2 reduced HR efficiency [28]. Also, this study showed that the protein expression of ligase IV decreased in response to Msh2 inhibition in HC11 cells. The results of this study are contrary to the finding that NHEJ efficiency was increased in Mlh1- and Msh2-deficient cells reported by Lin et al [29]. However, they are consistent with the study by Sible et al [30], which reduced HR and NHEJ frequencies in Msh2-deficient cells.

Gene editing technology is essential in the field of biotechnology, including research on gene function, the production of disease model animals, the production of therapeutic proteins, and the preservation of genetic diversity. As stated in the introduction, despite various studies related to the DNA repair pathway, the efficiency of successful gene editing is very low. Therefore, the development of efficient gene editing technology that improves the efficiency of HR is significant for the advancement of the field of animal biotechnology.

We highlight the complex interaction between MMR, NHEJ, and the HR pathway. In this study, inhibiting the Msh2 gene simultaneously decreased the expression of the NHEJ-related gene DNA ligase IV and the HR-related gene Rad51. Consequently, the reduced efficiency of HR-mediated knock-in due to Msh2 gene inhibition is attributed to the decreased expression of Rad51. Therefore, this study suggests that inhibiting the *Msh2* gene cannot be used to improve gene editing efficiency. In addition, considering the close association between HR and MMR, it is suggested that various studies on the regulation of MMR-related gene expression in HR-mediated knock-in systems are needed. Furthermore, since the Msh2 deficiency in this study reduced the HR-mediated gene editing efficiency, it seems that studying Msh2 overexpression should be supplemented. In conclusion, inhibiting Msh2 in HC11 cells downregulates HR-related Rad51 gene expression and inhibits gene editing efficiency.

## Figures and Tables

**Table 1:** List of primers used to construct the Msh2 expression siRNA vector

Gene	Primer name	Sequence (5′ → 3′)
**Oligonucleotide sequences in the Msh2 siRNA expression vector**		

Msh2	mMsh2 siRNA 1 BamHI	GATCCTTAGCAAGATGAACTTTGATTCAAGAGATCA AAGTTCATCTTGCTAAGCA
	mMsh2 siRNA 1 HindIII	AGCTTGCTTAGCAAGATGAACTTTGATCTCTTGAATC AAAGTTCATCTTGCTAAG
